# A first insight into the genotypic diversity of *Mycobacterium tuberculosis* from Rwanda

**DOI:** 10.1186/1472-6890-12-20

**Published:** 2012-11-06

**Authors:** James Gafirita, Alaine N Umubyeyi, Benon B Asiimwe

**Affiliations:** 1 Department of Laboratory Biomedical Sciences, Kigali Health Institute, P O Box 3286, Kigali, Rwanda; 2 Department of Science, National University of Rwanda, P O Box 56, Butare, Rwanda; 3 Department of Medical Microbiology, College of Health Sciences, Makerere University, P O Box 7072, Kampala, Uganda

## Abstract

**Background:**

*Mycobacterium tuberculosis* complex (MTC) is the causative agent of tuberculosis (TB). Globally, increasing evidence shows that in *M. tuberculosis,* transmission varies from strain to strain and that different strains exhibit a range of geographical and host specificities, pathogenicity, and drug susceptibility. Therefore rapid and accurate differentiation of the members of MTC is critical in guiding treatment and public health decisions. We carried out a study at different health units and the National Reference Laboratory in Rwanda identify *Mycobacterium tuberculosis* complex species prevalent in TB patients in Rwanda. We further characterized the isolates using spoligotyping in order to gain an insight into the strain diversity of drug resistant and susceptible isolates of *M. tuberculosis* in this setting.

**Methods:**

A total of 151 isolates from culture positive sputum samples were harvested, heat killed at 80°C for two hours, and then shipped to Makerere University College of Health Sciences, Uganda, for speciation and typing. Species identification was achieved by regions of difference (RD) analysis, while Spoligotyping was done to identify strain types.

**Results:**

Region of difference analysis identified all the 151 isolates as *M. tuberculosis*. Spoligotyping revealed predominance of the T2 family (58.3%, 88/151), with SIT 52 being the most prevalent strain (31.8%, 48/151). Among the 151 isolates, 64 (42.4%) were multidrug resistant (MDR) with 3 cases on mono-resistance. Of 94 retreatment cases, 48 (51.1%) were MDR and of 46 newly presenting cases 14 (30.4%) were MDR. There was a significant difference (p=0.01) in anti-TB drug resistance between new and retreatment cases in the sample. However, there was no significant relationship between HIV serostatus and the two major strain types SIT 52 (p =0.15and SIT 152 (p = 0.41).

**Conclusion:**

*Mycobacterium tuberculosis* is the most prevalent species of *Mycobacterium tuberculosis* complex in Rwanda, and SIT 52 (T2) the predominant strain. There is significantly more MDR in the retreatment cases but no significant difference was observed by HIV status in relation to any spoligotypes.

## Background

Together with other highly related bacteria, *Mycobacterium tuberculosis*, the major causative agent of tuberculosis (TB), forms a complex, the *Mycobacterium tuberculosis* complex (MTC), a single species as defined by DNA/DNA hybridization studies [1]. Other major members of the complex include *M. bovis,* which is mainly responsible for bovine TB, and *M. africanum,* the main causative agent of human TB in West Africa [2,3]. World over, many studies have shown that the propensity of spread of *M. tuberculosis* is dependent on strains types, and that these strains will not only be predominate in different settings but are also host specific [3-7]. DNA fingerprinting techniques in *M. tuberculosis* have made strain typing for epidemiology possible, thus it is now practical to predict transmission rates as well as identify and track strains associated with outbreaks [8], severe disease [9-11], and drug resistance [12].

In Rwanda, TB is one of the leading causes of mortality. Recent (2010) WHO data show a burden of 106 per 100,000 population [13]. Currently, the only data available on MTC in Rwanda focuses on drug resistance studies [14-16], and less is known about the prevalent species and strains, and how these relate with host demographic characteristics as well as drug resistance of the strains. Local studies on circulating MTC strains are important for comparison with the global *M. tuberculosis* population archived in various databases. Such knowledge enables a better understand the global traffic of common *M. tuberculosis* clades.

In the current study, genomic deletions also called regions of difference (RDs) were used to determine the predominant species of the MTC in TB patients in Rwanda. We analyzed samples brought in at the national tuberculosis reference laboratory (NRL) (located in Kigali) between March and September 2009. According to the National TB algorithm, NRL receives samples from all parts of the country, mainly: new cases with contact of known MDR patients; cases that had been on treatment for three months and remained sputum smear positive; and retreatment cases*.* Furthermore, we characterized our strain collection using spoligotyping, a robust and easy to perform technique that has found use in tracking TB epidemics, detecting new outbreaks, and better defining high-risk populations [17], so as to determine the genetic diversity of the strains from this locale. Following previous reports elsewhere of significant numbers of TB cases also co-infected with HIV [6,18], we investigated associations between the predominant spoligotypes and HIV sero-status of the patients as well as resistance to two key anti-tuberculosis drugs in this setting.

## Methods

### Ethical considerations

This study was approved by the Institutional Research and Ethics Committee of Kigali Health Institute, and Rwanda National Ethics Committee. Informed consent to participate in the study as well as permission to use isolates from samples provided were obtained from all enrolled participants. A materials transfer agreement was signed between the National Reference Laboratory (NRL) in Kigali, and the Department of Medical Microbiology at Makerere University College of Health Sciences, Uganda.

### Study setting

Rwanda has a population of about 320 persons per square Kilometer (2005 National Housing Census). Samples were obtained from sputum smear positive TB suspects presenting to several health units in Rwanda, between March and September 2010. These samples were brought to NRL in Kigali. According to the National TB algorithm, the NRL receives samples from all health centres in the country, mainly: new cases with contact of known MDR patients; cases that had been on treatment for three months and remained sputum smear positive; and retreatment cases*.* At the Hospitals and Health Centres where sampling was done, suspects provided a spot sputum sample on the first day, and were given another container to collect an early morning sample, and finally another spot sample was requested when the patient returned with the early morning sample. The sample with the highest ZN score was shipped to the National Reference Laboratory in Kigali using cetylpyridinium chloride-sodium chloride (CPC-NaCl) transport medium for on ward processing and culture. Suspects were also requested to provide 3mls of blood for HIV testing after pre-test counselling as per routine national policy for HIV testing in all TB patients in the country. Rapid screening for HIV was performed at the Hospitals and Health Centres that received the patients. All the HIV positive patients received post-test counselling and were referred to national HIV treatment centres for professional health care. Demographic data for each patient sample, consisting of age, sex, and TB treatment history were also obtained.

### Sample processing

At the NRL, about 5mls of specimen were homogenized by digestion for 1 minute at room temperature with 1 ml of N-acetyl L-cysteine (NALC, 25mg/ml) in phosphate buffer (pH 6.8) and vortexed with several 4 mm glass beads for 30 seconds. A 5 ml aliquot was decontaminated using 1% NaOH [19] and concentrated at 4000g for 15 minutes. The sediment was then reconstituted to 2.5 mls, using phosphate buffer pH 6.8, to make the inoculum for smears and cultures. Colonies were harvested in 400μl of sterile Tris-EDTA (TE) buffer, heat inactivated at 80°C for two hours and then shipped to the Department of Medical Microbiology at the College of Health Sciences, Makerere University, for identification and typing.

### Culture and drug susceptibility testing

Sediments were cultured on Lowenstein-Jensen medium (L-J), incubated at 37°C and read weekly for growth for a maximal duration of 10 weeks. Positive cultures were subjected to Ziehl-Neelsen (ZN) staining for confirmation of mycobacterial growth, and isolates were later confirmed as MTC at the molecular level by a previously described PCR typing panel [4]. Drug Susceptibility Testing (DST) was performed by the indirect proportion method on L-J media at the following drug concentrations: rifampicin, 40μg/ml and isoniazid, 0.2μg/ml as recommended elsewhere [20]. For all test panels, drug susceptible strain (H37Rv) and specific drug resistant strains (TMC 303 for isoniazid and TMC 331 for rifampicin) internal controls were included.

### DNA extraction

Cultures with ample growth were harvested, isolates heat killed for 2 hours and DNA extracted by the phenol-chloroform method using standard protocols [21]. For cultures that did not have ample harvests, heat killed isolates were used directly for PCR in subsequent analyses.

### RD analyses and spoligotyping

All the target genomic loci were previously well characterized [22,23]. Strains were analyzed for presence of the MTC specific 16S rRNA gene, and then RD9 (deleted in *M. africanum* but present in *M. tuberculosis*), as well as TbD1 (a *M. tuberculosis* specific deletion that is intact in *M. africanum*) using previously described PCR methods which detail primer sequences and amplification conditions [4,24]. Standard spoligotyping [25] was performed using a commercially available kit (Isogen Bioscience BV, Maarssen, The Netherlands) following manufacturer’s instructions.

### Data analysis

Spoligotypes were analyzed by the BioNumerics software, version 5.0 (Applied Maths, Kortrijk, Belgium) as character types. Binary outcomes were fed into the international spoligotyping database of the Pasteur Institute of Guadeloupe [17], which provides information on the spoligotype international type (SIT) distributions of *M. tuberculosis* spoligotypes worldwide. Statistical associations between strain types, drug susceptibility and HIV sero-status were generated by Stata 10 using the Pearson’s chi-square test, and a P value of <0.05 was considered evidence of a significant difference.

## Results

### Study population

Samples from 153 patients were brought to the NRL between March and September 2009 for culture, with 39 of the patients providing more than one sample for internal control, but these duplicate samples were not considered in the final statistical analysis. Furthermore, two isolates did not amplify for the 16srRNA locus even on repeat analysis and were thus considered atypical mycobacteria and excluded from further analysis. Therefore, only isolates from 151 patients were assayed in this study. Ninety of the 151 (59.6%) of the isolates were from male patients. The sample median age was 36 (Interquartile range [IQR] 28, 48). Stratification according to age showed that 70 (49.6%) of the patients were between 18 and 35 years old (youths) while 71 (50.4%) were over 35 years of age.

### Species identification

From the resulting PCR patterns for the three targeted RD loci, all the 151 isolates were identified as *M. tuberculosis* (all deleted at the TbD1 locus), with consistent amplification for RD9, a region that is invariably deleted from all *M. africanum* → *M. bovis* lineage strains as previously shown elsewhere [22].

### Spoligotypes

**Figure 1:**
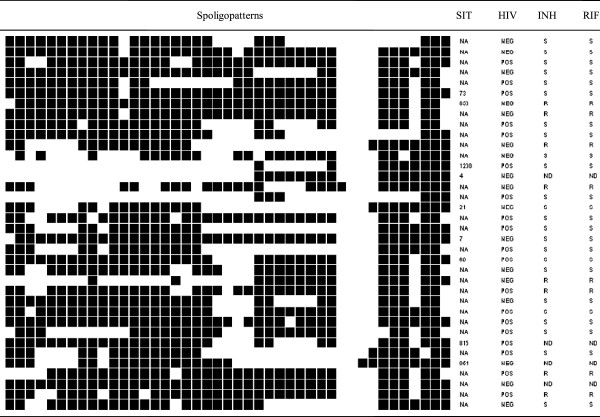
**Spoligotype patterns of non-clustered *M. tuberculosis *strains in the study (N=36). **SIT, spoligotype international type; filled boxes represent positive hybridization while empty boxes represent absence of spacers; label defines the lineage/sub lineage; NA, not available in SpolDB4.0; HIV, sero-status of the patients; INH, resistance to isonaizid; RIF, resistance to rifampicin; S, sensitive; R, resistant; ND, not determined.

To determine the strain lineages present in the sample, the 151 isolates were spoligotyped and binary outcomes compared with those existing in SpolDB4 so as to assign spoligotype international type (SIT) designations. A total of 115 isolates (76.2% of the sample) were grouped into 17 clusters (2 to 48 isolates per cluster), while the remaining 36 (23.8%) of the strains did not cluster. Of these 36 strains that did not cluster, 27 did not exist in the SpolDB4.0 data base, hence represented the true orphans in the study sample. The remaining nine of the un clustered isolates were all present in SpolDB4 with labels SIT 73 (T2-T3), SIT 853 (T2), SIT 1208 (H1), SIT 4 (LAM 3/S Convergent), SIT 21 (CAS_KILI), SIT 7 (T1), SIT 60 (LAM 4), SIT 815 (LAM11_ZWE) and SIT 954 (CAS_DELHI). The associated drug susceptibility patterns for the un clustered isolates as well as HIV sero-status of the corresponding patients for each isolate are indicated in Figure 1.

**Figure 2:**
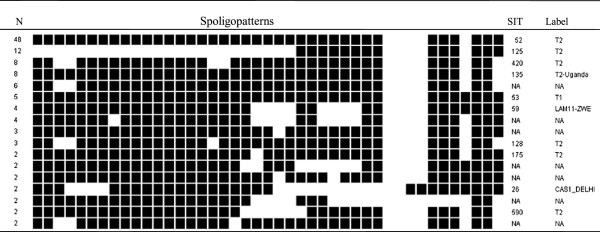
**Spoligotype patterns of 115 clustered *M. tuberculosis *strains in the study. **N, number of isolates per cluster; SIT, spoligotype international type; filled boxes represent positive hybridization while empty boxes represent absence of spacers; label defines the lineage/sub lineage; NA, not available in SpolDB4.0.

Among the 17 clusters, only two included more than ten isolates each and were defined as major spoligotypes, while minor spoligotypes, in this study, were defined as SITs that contained two to eight isolates per cluster. The two major shared spoligotypes in our sample were SIT 52 (T2) with 48/151 (31.8%) and SIT 125 (T2) with 12/151 (7.9%) of the isolates. Other significant clustered spoligotypes in the sample were SIT 420 (T2) and SIT 135 (T2-Uganda) with eight strains each (Figure 2). Furthermore seven clusters, ranging from two to six strains per cluster, formed a total of 20 strains and were not yet defined in SpolDB4.0. Among all the clustered strains, 83 of 115 (72%) were identified in SpolDB4 as T2, while a further 15 strains that were not identified in SpolDB4 also lacked hybridization to either spacer 40 or both 40 and 43, characteristic of the T2 Euro-American lineage of strains previously erroneously identified in Uganda as *M. africanum* genotypes Uganda II and I respectively [26] but later termed *M. tuberculosis* Uganda genotype strains [4].

### Drug susceptibility patterns

Susceptibility testing results for the two key anti-tuberculosis drugs (isoniazid and rifampicin) showed that 67 isolates were susceptible to both drugs, three isolates were monoresistant (two to rifampicin and one to isonaizid), resistance to isoniazid was 65/151 (43%), and that to rifampicin was 66/151 (43.7%), while 17 cases did not have interpretable susceptibility results. Sixty four of the 65 isonaizid resistant strains were also rifampicin resistant hence MDR. Of the 151 patients in the study, 94 were retreatment cases, of the 46 new patients, 3 new cases were MDR know contact patients whereas 43 new patients were on treatment for three months and remained microscope smear positive, while treatment history for 11 patients could not be established. Among the retreatment cases, 48/94 (51.1%) were MDR, while 13/46 (28.3%) of the new cases were MDR (p = 0.01). A summary of patient demographic characteristics and associated drug susceptibility pattern is shown in Table 1.

**Table 1:** Patient demographic characteristics and associated drug susceptibility pattern

**Demographic characteristics**		**Total**	**Sensitive**^**a**^	**Resistant**		
				**INH**^**b**^	**RIF**^**c**^	**MDR**^**d**^
Number of strains		151	67 (44.4%)	1 (0.7%)	2 (1.3%)	64 (42.4%)
Sex	Male	95	47 (49.5%)	1 (1.1%)	1 (1.1%)	34 (35.8%)
	Female	56	20 (35.7%)	0	1 (1.8%)	30 (53.6%)
Treatment history	New cases	46	28 (60.9%)	0	0	12 (26.1%)
	Retreatment	94	39 (41.5%)	1 (1.1%)	2 (2.2%)	48 (51.1%)
	Unknown	11	2 (18.2%)	0	0	0
HIV status	Positive	69	34 (49.3%)	0	2 (2.9%)	30 (43.5%)
	Negative	76	35 (46.1%)	1 (1.3%)	0	32 (42.1%)
	unknown	6	0	0	0	0

^a^Sensitive indicates isolates susceptible to both isoniazid and rifampicin; ^b^Resistance to isonaizid only, ^c^resistance to rifampicin only, ^d^resistance to both isonaizid and rifampicin. Seventeen isolates did not have complete susceptibility testing results and were excluded from the table.

Analysis of drug resistance in the major clusters revealed that SIT 52 (T2) with 48 strains had 34/65 (52.3%) of the total isoniazid resistant strains in the sample. Furthermore, this strain type had 35/66 (53%) of the rifampicin resistant strains and 34/64 (51.3%) of the MDR isolates. SIT 125 (T2), on the other hand, had eight of its12 strains resistant to both rifampicin and isoniazid, hence MDR. Categorization of the patients into retreatment and new cases within the two major spoligotypes revealed that 35/48 strains in SIT 52 were retreatment cases while 11 of the 12 cases in SIT 125 were retreatment. There were no significant statistical associations between genotypes and drug resistance*.* The relationship between the different spoligotypes in the non clustered strains and resistance to rifampicin and isoniazid is summarized in Figure 1.

### HIV sero-status and associated spoligotypes

In the sample analyzed, 69 patients (45.7%) were HIV sero-positive, 76 (50.3%) sero-negative, while 6 (4%) did not have test results hence their status unknown. Of the 69 sero-positive cases, 42 (60.9%) were TB retreatment cases while 52/76 (26.3%) of the sero-negative cases were retreatment. An analysis of the drug susceptibility pattern of isolates from the 69 HIV sero-positive individuals showed that 31 had strains resistant to isoniazid, 32 to rifampicin while 30 (43.5%) were MDR isolates. Analysis of the two major spoligotypes above (SIT 52 and SIT 152) vs. HIV sero-status of patients showed that 19 of the 48 SIT 52 strains (39.6%) were from HIV positive patients while 26/48 (54.2%) strains were from HIV negative patients (p =0.15). Likewise 7 of the 12 SIT 152 strains (58.3%) were isolated from HIV positive patients while 5/12 (41.7%) were from HIV negative patients (p = 0.41). There was no statistical relationship between HIV sero-status of the patients and any particular spoligotypes pattern. The sero-status of the patients carrying un clustered strains in the study is shown in Figure 1.

## Discussion

This, to the best of our knowledge, is the first report describing the species and strain diversity of *M. tuberculosis* complex isolates from TB patients in Rwanda. Characterization of prevailing *M. tuberculosis* strains focusing on different geographical levels is important for locating the origin, evolution and spread dynamics of particular *M. tuberculosis* clones, which is often difficult to be identified by traditional epidemiological investigations. In low-resource, high-disease burden settings, it is critical to identify circulating strains in order to understand the dynamics of spread of the causative agent. In Rwanda, there is no data about the species and strains of *M. tuberculosis* circulating in the country. This report, therefore, will provide baseline data for future country-wide molecular epidemiological studies to understand transmission dynamics of TB.

Regions of Difference (RD) analysis using 16S-rRNA, RD9 and TbD1 loci showed that all the strains investigated were characterized by presence of both 16S-rRNA and RD9 loci, and deletion in the TbD1 regions, a pattern confirming that they all were *M. tuberculosis* strict sense. Most studies in the East African region have reported predominance of *M. tuberculosis*[4,26,27], while most *M. africanum* strains isolated to date are from West Africa [2,7,28,29].

A majority (68.2%) of the spoligotypes obtained in this study belong to previously identified shared spoligotype international types (SITs). A significant proportion of the total isolates (48/151, 31.8%) belonged to SIT 52, while only 8/151 (5.3%) were SIT 135, a strain type commonly seen in Uganda. SIT 52 was found to be 7.6% (26/344) of isolates in a study in Central Uganda [18] and 4.8% (6/125) of isolates from South Western Uganda [30], while not a single strain of this type was seen in a collection of 130 isolates from Northern Tanzania [31]. Generally, this genotype together with the related SIT 135 and SIT 128 are known to be the commonest strain types causing TB in Central African human host populations [4].

The 151 isolates in the study show 53 different spoligopatterns, displaying a wide diversity of the spoligotypes in this collection. It is known that the structure of the TB populations is determined by geography, demography, and human migration. The large diversity of strains observed in this study may be attributed to increased transborder human movement in this region due to a large influx of former refugees from different neighboring countries in the last 15 years. Additionally, true orphan spoligotypes accounted for only 17% of all the spoligotypes in this study, this low percentage further supporting the hypothesis of increased recent human traffic in this setting, since countries with a history of isolation have been shown to have a large number of new spoligotypes, which is not the case in this scenario [32].

Spoligotyping identified T2 to be the most predominant family of strains in Rwanda, accounting for up to 55.0% (83/151) of the total sample (Figures 1 and 2). Results from a previous molecular study of recurrent TB in Rwanda by spoligotyping and mycobacterial interspersed repetitive unit variable number of tandem repeat (MIRU-VNTR) typing did not show species and strain types in the collection [16] hence we cannot compare the two studies. Findings from the current study, however, are in agreement with the previous data from Uganda, in which two studies showed predominance of T2 family, the first having been conducted at the National referral hospital, Kampala, in which 67% of the isolates were T2 [33] and the second a systematic community based study in Rubaga, one of the divisions of Kampala, which reported 70% isolates being of the T2 family [18]. This result is in further agreement with those elsewhere reporting predominance of single genotypes in the respective populations across Africa [2,6,7,28,34]. Collectively, these results depict a tendency for local genotypes that are well established to form a larger proportion of circulating strains compared to others as previously postulated [3,35]. Since our sample collection may not reflect a national picture, a future national survey could genotype all isolates so as to give a clear situation of strain types as well as transmission pattern in this locale.

In Rwanda, the most recent national anti-tuberculosis drug resistance survey (2002–2005) on 616 new cases [14] showed that 6.2% of the isolates were resistant to isoniazid, 3.9% to rifampicin and 3.9% were multi-drug resistant. In neighboring Uganda, a much earlier national survey (1996–1997) showed that of 586 patients, resistance to isoniazid was 6.7 %, that to rifampicin was 0.8% while MDR was 0.5% [36]. The current study tested 46 new TB case, 94 retreatment cases and 11 cases with no known history. Overall MDR was 42.4%, a very high increase, most likely attributed to the high proportion of retreatment cases (94/115) in our study population as opposed to new TB cases in the previous studies.

## Conclusion

Mycobacterium tuberculosis is the most prevalent species of *Mycobacterium tuberculosis* complex in Rwanda, and SIT 52 (T2) the predominant strain. There is significantly more MDR in retreatment cases but no significant difference was observed by HIV status in relation to any spoligotypes.

## Competing interests

The authors declare that they have no competing interests.

## Authors’ contributions

JG participated in the planning of the study, acquisition of samples, culture and isolation of mycobacteria and molecular analysis of isolates; BBA participated in planning of the study, training JG, supervision of the molecular assays, data analysis and drafting of the manuscript. ANU participated in data collection, seeking ethical clearance and material transfer agreement, supervision of the work, and drafting of the manuscript. All authors read and approved the final manuscript.

## Pre-publication history

The pre-publication history for this paper can be accessed here:

http://www.biomedcentral.com/1472-6890/12/20/prepub
